# Protective mechanism of 1-methylhydantoin against lung injury induced by paraquat poisoning

**DOI:** 10.1371/journal.pone.0222521

**Published:** 2019-09-27

**Authors:** Bo Liu, Annan Chen, Jinyi Lan, Lei Ren, Yifan Wei, Lina Gao

**Affiliations:** 1 The 3^rd^ Clinical Department of China Medical University, Shenyang, Liaoning, China; 2 School of Public Health, China Medical University, Shenyang, Liaoning, China; 3 School of Forensic Medicine, China Medical University, Shenyang, Liaoning,China; University of Pécs Medical School, HUNGARY

## Abstract

Paraquat (PQ), one of the most widely used herbicides worldwide, causes severe toxic effects in humans and animals. 1-methylhydantoin (MH) is an active ingredient of Ranae Oviductus, which has broad pharmacological activities, e.g., eliminating reactive oxygen species and inhibiting inflammation. This study investigated the effects of MH on lung injury induced by PQ. A PQ poisoning model was established by intragastric infusion of PQ (25 mg/kg), and the control group was simultaneously gavaged with the same dose of saline. The MH group was intraperitoneally injected with 100 mg/kg once per day after intragastric infusion of PQ (25 mg/kg) for five consecutive days. All animals were sacrificed on the sixth day, and the lung tissues were dissected for metabolomics analysis. The lactate dehydrogenase (LDH) activity, superoxide dismutase (SOD) activity, TNF-α and malondialdehyde (MDA) content were determined according to the instructions of the detection kit. Compared with that in the control group, the content of LDH, TNF-α and MDA in the lung tissue of the PQ group was significantly higher, and the activity of SOD in the lung tissue was significantly lower (all p<0.05). Compared with that in the control group, the content of LDH, TNF-α and MDA in the MH group was significantly higher, and the activity of SOD was significantly lower (all p<0.05). However, the differences in SOD activity, LDH activity between the PQ and MH groups were not statistically significant (all p > 0.05). There were significant differences in MDA and TNF-α content between the PQ group and MH group (all p<0.05). MH decreased the production of malondialdehyde and TNF-α to protect against the lung injury caused by PQ poisoning, but it had no significant effect on the activity of LDH and SOD. There were significant differences in metabolomics between the MH group and the PQ poisoning group, primarily in bile acid biosynthesis and metabolism of cholesterol, nicotinate, nicotinamide, alanine, aspartate, glutamate, glycine, threonine, serine, phenylalanine and histidine. Therefore, this study highlights that MH has non-invasive mechanisms and may be a promising tool to treat lung injury induced by PQ poisoning.

## Introduction

Paraquat (PQ) is a herbicide that is widely used in agriculture and is extremely toxic to humans and animals [1]. There are no specific antidotes for PQ poisoning, and the patient mortality rate is as high as 90% after PQ poisoning [2]. PQ exerts its toxic effects primarily through its redox cycle through the production of superoxide anions in organisms; thus, leading to an imbalance in the redox state of the cell and causing oxidative damage and cell death [1]. Although there is no treatment for PQ poisoning, several studies have suggested antioxidant therapy as a viable alternative. For example, antioxidants such as vitamin C [3][4] and lysine acetylsalicylate [5] have been shown to be useful in the treatment of PQ toxicity.

Metabolomics, also often referred to as "metabolic profiling," is the systematic profiling of metabolites and their temporal changes in biofluids or tissues of organisms [6]. Metabolomic profiling of biological systems has the powerful ability to provide biological understanding of the metabolic functional states responding to environmental factors or other perturbations [7].

Ranae Oviductus is known to have a wide variety of pharmacological effects, e.g., anti-inflammatory, anti-fatigue and anti-oxidant activity [8,9]. However, Ranae Oviductus is obtained from the Northeast forest frog, a wild animal found in the mountainous area of northeast China, including the Changbai mountains and most of the little Xingan ridge. The northeast frog is found only in China and is listed as a vulnerable species.

1-methylhydantoin (MH) is the active ingredient in Ranae Oviductus, which is used to evaluate the quality of Ranae Oviductus in the Chinese Pharmacopoeia (Chp; 2015 edition). A process to synthesize MH has been developed [10,11]. MH can be used as a supplement for Ranae Oviductus, and it also has broad pharmacological activities. Li Wei et al. [12] have found that MH can eliminate reactive oxygen species (ROS); can inhibit proliferation and induce apoptosis in colon cancer SW480 cells *in vitro*; and can block the cell cycle in G0/G1 phase. Han D et al. [13] have found that MH inhibits airway inflammation and relaxes bronchial smooth muscle; thus, resulting in antiasthmatic and antitussive effects. You J et al. [14] have found that MH has anti-depressive effects. Overall, MH can be used as an antioxidant and inhibitor of inflammation. Moreover, the main mechanism of PQ poisoning is the massive production of superoxide anions, thus, causing oxidative damage and leading to cell death [15].

**Fig 1:**
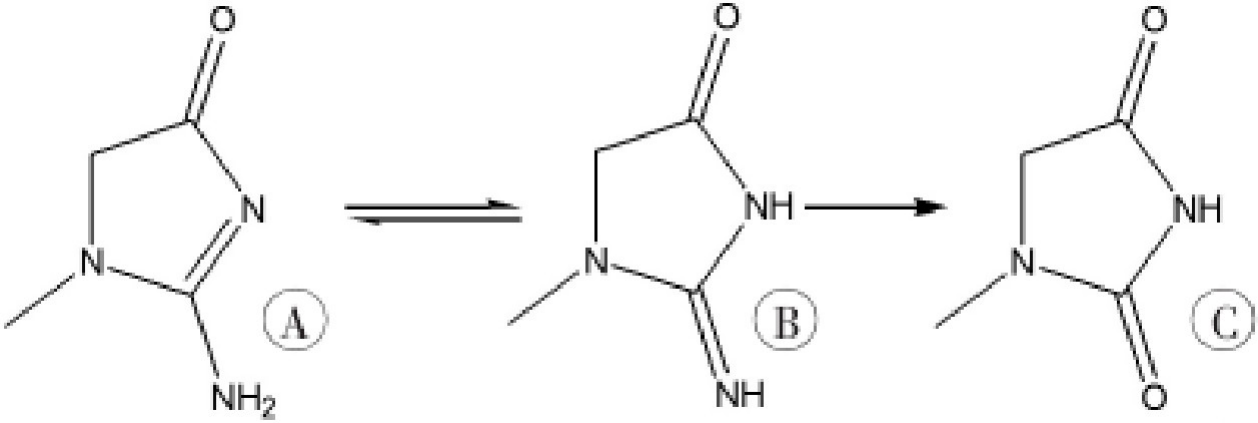
The metabolic pathway of creatinine (A. creatinine; B. geometric isomer of creatinine; C.1-methylhydantoin).

PQ poisoning can result in multiple organ failure that primarily affects the lungs, kidneys, liver and nervous system. The kidneys, as the main detoxification organ, encounter very high concentrations of PQ during the body’s process of PQ elimination, thus leading to acute kidney injury [16]. Therefore, protecting kidney function is also an effective treatment for PQ poisoning. Some studies have found that hydantoin derivatives can be used as a treatment for renal failure [17–19], and we have revealed that the 5-hydroxy-1-methylhydantoin (HMH), the analogue of MH, a mammalian creatinine metabolite and an intrinsic antioxidant [17–19], protects against PQ toxicity [20,21]. MH is a metabolite of creatinine formed through deamination by deaminase produced by microorganisms in the gastrointestinal tract [22,23], as shown in Fig 1. Although some studies have found that MH can be used not only as an antioxidant to inhibit inflammation but also as a treatment for renal failure, the effects of MH on the PQ toxicity are unclear. PQ induces pulmonary fibrosis, and the lungs are the target organ after PQ administration. This study was conducted to explore the protective mechanism of MH against lung injury induced by PQ poisoning, through investigation of metabolomics; the activity of lactate dehydrogenase (LDH) and superoxide dismutase (SOD); and the content of malondialdehyde (MDA) and tumor necrosis factor-α (TNF-α).

## Materials and methods

### Chemical and reagents

MDA was purchased from Shanghai Yiyan Biotechnology Co., Ltd. (Shanghai, China), and PQ was purchased from Macklin Shanghai Biotechnology Co., Ltd. (Shanghai, China). BCA protein assay kit and total SOD activity detection kit was purchased from Beyotime Biological Reagent Co., Ltd. (Shanghai, China), an LDH activity detection kit was purchased from Solarbio Life Science Company (Shanghai, China), and MDH and TNF-α detection kits were purchased from BestBio Biological Reagent Co., Ltd. (Shanghai, China). Methanol, water, acetonitrile and formic acid were all LC-MS grade and were purchased from Thermo Fisher Technology (USA) Co., Ltd.

### Animal experiments and sample collection

Thirty Kunming male mice of Specific pathogen Free grade were used. Mice were 4 weeks old and had body weights of 30 g±2 g. They were purchased from Liaoning Changsheng Biotechnology Co., Ltd. Procedures were performed under animal certificate number SYXK (Liaoning Province) 2018–0008. The 30 mice were divided into a control group, PQ poisoning group and MH group (with ten mice randomly allocated to each group). The PQ poisoning group was given PQ at 25 mg/kg by gavage. The control group was given the same amount of normal saline by gavage. The MH group received a 100 mg/kg intraperitoneal injection after intragastric infusion of PQ (25mg/kg). The MH group received intraperitoneal injections at the same time every day for five consecutive days. All mice were sacrificed on the 6th day for collection of lung tissue specimens. Briefly, at the day of sacrifice, animals were euthanized according to NIH ARAC guidelines for euthanasia of rodents using carbon dioxide. Mice were euthanized by trained personnel via source of compressed gas in their home cage. After checking each mouse for lack of respiration and faded eye color, we confirmed their death. CO2 flow was maintained for a minute after respiration ceases. Animal handling and care conformed to the guidelines of current international laws and policies (National Institutes of Health Guide for the Care and Use of Laboratory Animals, Publication No. 85–23, 1985, revised 1996; Animal protection law of the People's Republic of China, publication 2009) and were approved as ethical by the Administration Committee of Experimental Animals at the Laboratory Animal Center of China Medical University (CMU-2018072).

### Quantification of SOD, MDA and LDH in lung homogenates

Levels of SOD, MDA and LDH were assessed in lung homogenates using detection kits, according to the manufacturer’s instructions.

### Detection of TNF-α in lung homogenates

An ELISA kit was used to measure the TNF-α levels. According to the kit instructions, after the preparation of the plates, 100 μl of buffer solution was added to the sample, and then the solution was maintained at room temperature for 2 hr. The samples were aspirated and washed in two steps, and then 200 μl of substrate solution was added to each well and incubated at room temperature for 2 hr. Then 100 μl of antibody solution was added to each well, and the plates were incubated at room temperature for 1 hr. After the aspiration/wash in two steps was repeated, 200 μl of substrate solution was added to each well and incubated at room temperature for 20 min. Finally, 500 μl of stop solution was added to each well. The optical density of each plate was determined with an ELISA reader set at 450 nm wavelength [24].

### Instrument conditions

Chromatographic conditions: Samples were injected onto an Accucore HILIC column (100 mm×2.1 mm, 2.6 μm) at a flow rate of 0.3 mL/min, and the column temperature was set at 40°C. The eluents with positive polarity mode were eluent A (0.1% FA in 95% ACN, 10 mM ammonium acetate) and eluent B (0.1% FA in 50% ACN, 10 mM ammonium acetate). The eluents with negative polarity mode were eluent A (95% ACN, 10 mM ammonium acetate, pH 9.0) and eluent B (50% ACN, 10 mM ammonium acetate, pH 9.0). The chromatographic gradient elution procedure was optimized as follows: 0–1 min, A%:B% = 98:2; 17–17.5 min, A%:B% = 50:50; and 18–20 min, A%:B% = 98:2.Mass spectrometry conditions: A Q-Exactive HF-X mass spectrometer was selected in a scan range of 100–1500 m/z by using MS/MS scan for data-dependent full scans, operated in positive and negative polarity mode with a spray voltage of 3.2 kV, capillary temperature of 320°C, sheath gas flow rate of 35 arb and aux gas flow rate of 10 arb.

### Metabolite extraction

Tissues (100 mg) were individually ground with liquid nitrogen, and 100 μL of homogenate was resuspended with precooled 100% methanol (-20°C) and thoroughly vortexed. The samples were incubated at -20°C for 60 min and then centrifuged at 14000 g at 4°C for 15 min. Then the supernatants were transferred to a fresh microcentrifuge tube and dried under vacuum in a centrifugal evaporator. The dried metabolite pellets were redissolved in 80% methanol and analyzed by LC-MS/MS.

### Untargeted metabolomics analysis

After metabolic information collection and data preprocessing, the resulting matrix was imported into SIMCA-P (version 13.0, Umetrics, Sweden) for unsupervised principal component analysis (PCA) and supervised partial least-squares-discriminant analysis (PLS-DA). PCA was used to obtain a preliminary overview of grouping trends, and PLS-DA was used to identify the potential biomarkers between the groups [25–27] (the NS group vs. the PQ poisoning group, and the PQ poisoning group vs. the MH group). Discriminating metabolites were identified with variable influence on the projection (VIP) plots (99% confidence) [27]. For each multivariate model, the calculated R^2^ value reflected the goodness of fit. The parameter Q^2^ of PLS-DA represented the predictive ability of the model: a Q^2^ value approaching 0.5 indicated a good model.

Differential variables correlating with PQ toxicity and MH pharmacology were screened as follows: first, the VIP value was required to be greater than 1.0, the contribution for grouping. Second, to decrease the probability of false positives, an adjusted P value from the nonparametric Mann-Whitney U test (PASW Statistics 19, SPSS Inc., Chicago, United States) was determined and was required to be lower than 0.05[28]. Third, the value of the area under the receiver operating characteristic (AUC-ROC), an evaluation parameter for classification performance, was calculated in PASW Statistics 19 (SPSS Inc., Chicago, IL, United States), and the variables were discarded when AUC-ROC ≤ 0.75. Moreover, the classification performance was considered excellent when AUC-ROC > 0.9[29]. Metabolite heat maps were produced in MultiExperiment View (Version 4.9.0). The changes in metabolites in each group were determined through a volcano map. The Kyoto Encyclopedia of Genes and Genomes (KEGG) pathway database was used to perform enrichment analysis of differential metabolites and pathway analysis.

## Results

### SOD activity, LDH activity and MDA content

Oxidative stress is a key factor that leads to mitochondrial damage. Oxidative stress results in a significant increase in LDH and MDA levels as well as a decrease in the SOD level. As shown in Table 1, compared with those in the control group, the content of LDH and MDA in the lung tissue of the PQ group was significantly higher, and the activity of SOD in the lung tissue was significantly lower (LDH (U/mg protein) 19.23±1.8 vs. 8.91±1.76; MDA (mol/mg protein) 0.535±0.064 vs. 0.13±0.05; and SOD (U/mg protein) 172.2±2.54 vs. 272.8±2.54 U/mg protein). Compared with that in the control group, the content of LDH and MDA in the MH group was significantly higher, and the activity of SOD was significantly lower (LDH (U/mg protein) 15.06±4.74 vs. 8.91±1.76; MDA (mol/mg protein) 0.299±0.046 vs. 0.13±0.05; and SOD (U/mg protein) 177.8±1.10 vs. 272.8±2.54). However, the differences in SOD activity and LDH activity between the PQ and MH groups were not statistically significant (all P > 0.05). There were significant differences in the MDA content between the PQ group and MH group (MDA (mol/mg protein) 0.535± 0.064 vs.0.299±0.046).

**Table 1:** LDH, SOD and MDA levels in lung tissue in different groups.

groups	Number of animals	LDH(U/mgprot)	SOD(U/mgprot)	MDA(μmol/mgprot)
control group	10	8.91±1.76	272.8±2.54	0.13±0.05
MH group	10	15.06±4.74^a^	177.8±1.10^a^	0.299±0.046^ab^
PQ group	10	19.23±1.8^a^	172.2±2.54^a^	0.535±0.064^a^

Note: The MH group was intraperitoneally injected at a dose of 100 mg/kg after paraquat gavage. The PQ poisoning animal model was prepared by one-time intragastric administration of 25 mg/kg PQ. Compared with the control group, ^a^P<0.05, compared with the PQ group, ^b^P<0.05.

### TNF-α levels in different groups

**Fig 2:**
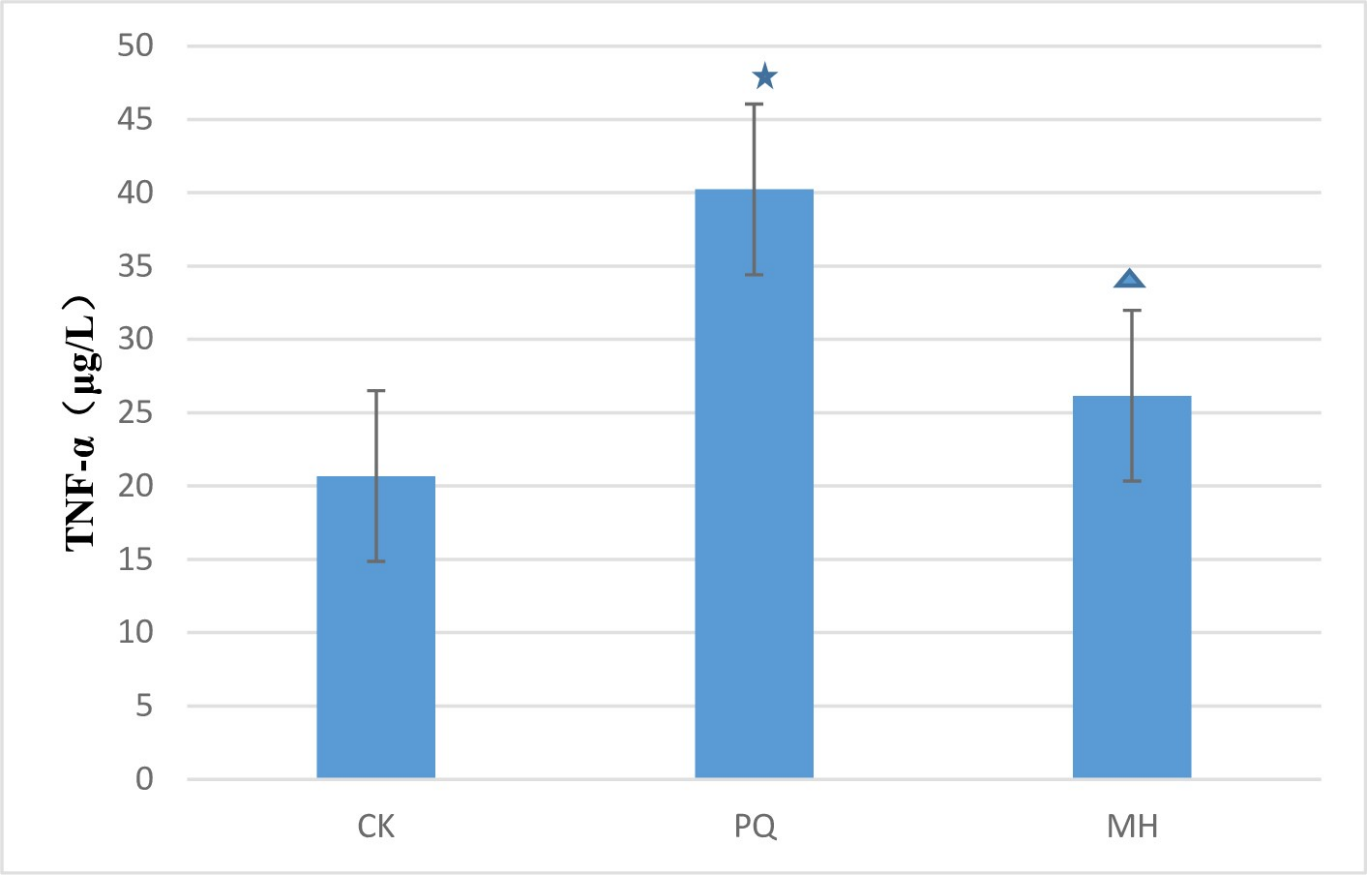
Comparison of lung TNF-α level in the studied groups. The statistical analysis was performed by one-way ANOVA and p < 0.05 was considered as statistically significant. Data represent the mean ± SEM of 6 animals. p*<*0.05 in relation to CK group; p *<*0.05 in relation to PQ group. CK means the control check group, PQ means the paraquat group, MH means the MH group.

As shown in Fig 2, the level of TNF-α differed between the MH and the PQ group. There was a clear difference between the MH group and the control group, and between the PQ group and the control group. We speculated that MH might decrease inflammation in the lung tissue after PQ poisoning.

### Metabolomics results

**Fig 3:**
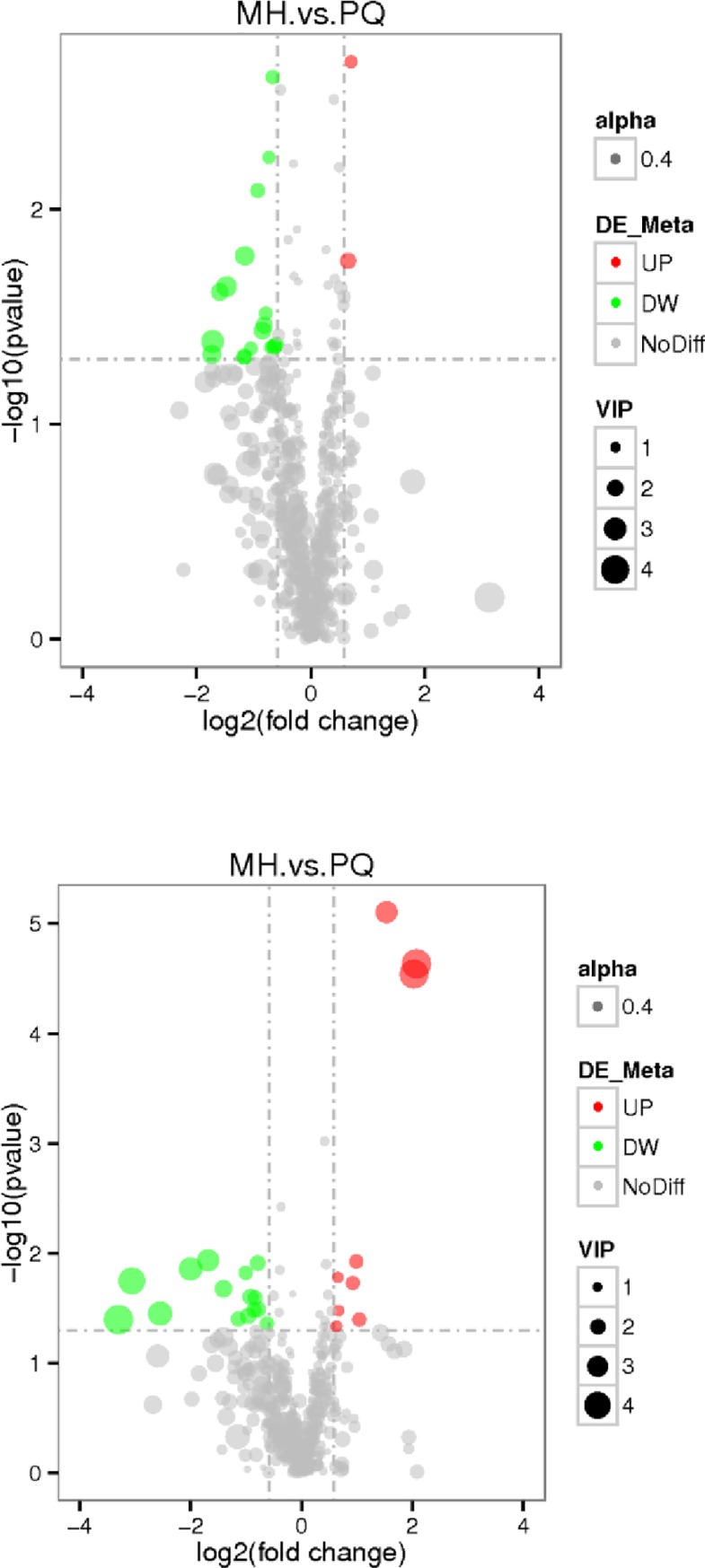
The volcano map of differential metabolites (the above figure represents the results were obtained under the MS positive ion model, and the below figure represents the results under the MS negative ion mode. Red represents up-regulation, and green represents down-regulation in the MH group, gray represents there is no distinguished difference between the MH group and the PQ goup, and VIP represents the importance projection value of this substance obtained in the PLS-DA model compared in this group).

The changes in metabolites under the MS positive and negative ion mode were observed in the volcano map of different metabolites, and the results are shown in Fig 3. Red represents metabolites up-regulated as compared with the PQ group, green represents metabolites down-regulated as compared with the PQ group, and gray represents metabolites with no difference between the PQ group and MH group. The VIP value represents the importance projection value of the metabolites.

**Fig 4:**
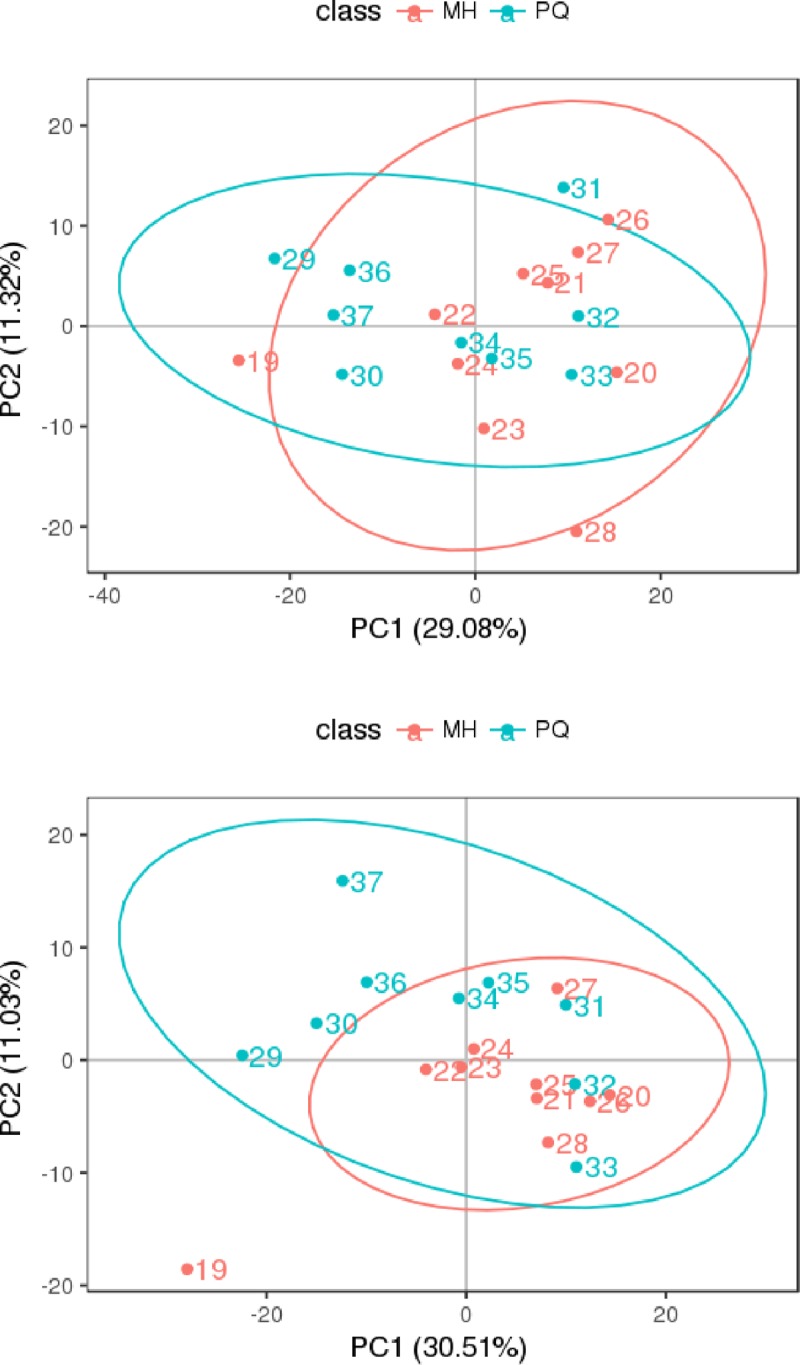
PCA score results of mice lung samples, the above figure represents the result was obtained under the MS positive ion mode, and the below figure represents the result was obtained under the MS negative ion mode.

**Fig 5:**
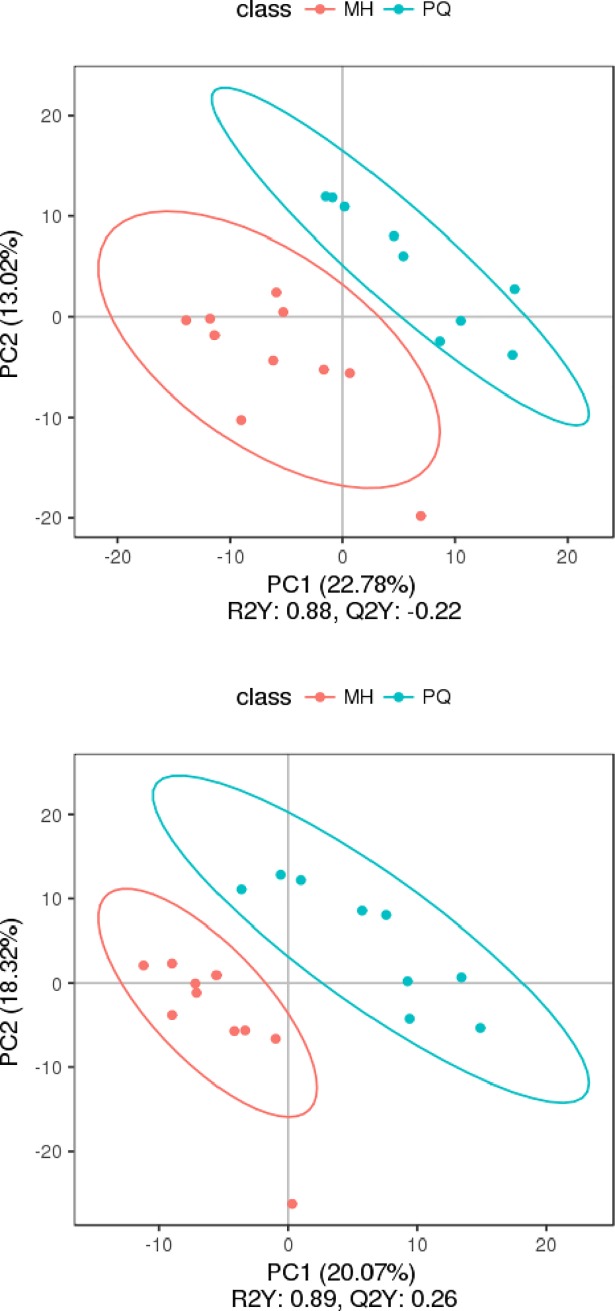
PLS-DA score, the above figure represents the results were obtained under the MS positive ion mode, and the below figure represents the results were obtained under the MS negative ion mode.

**Fig 6:**
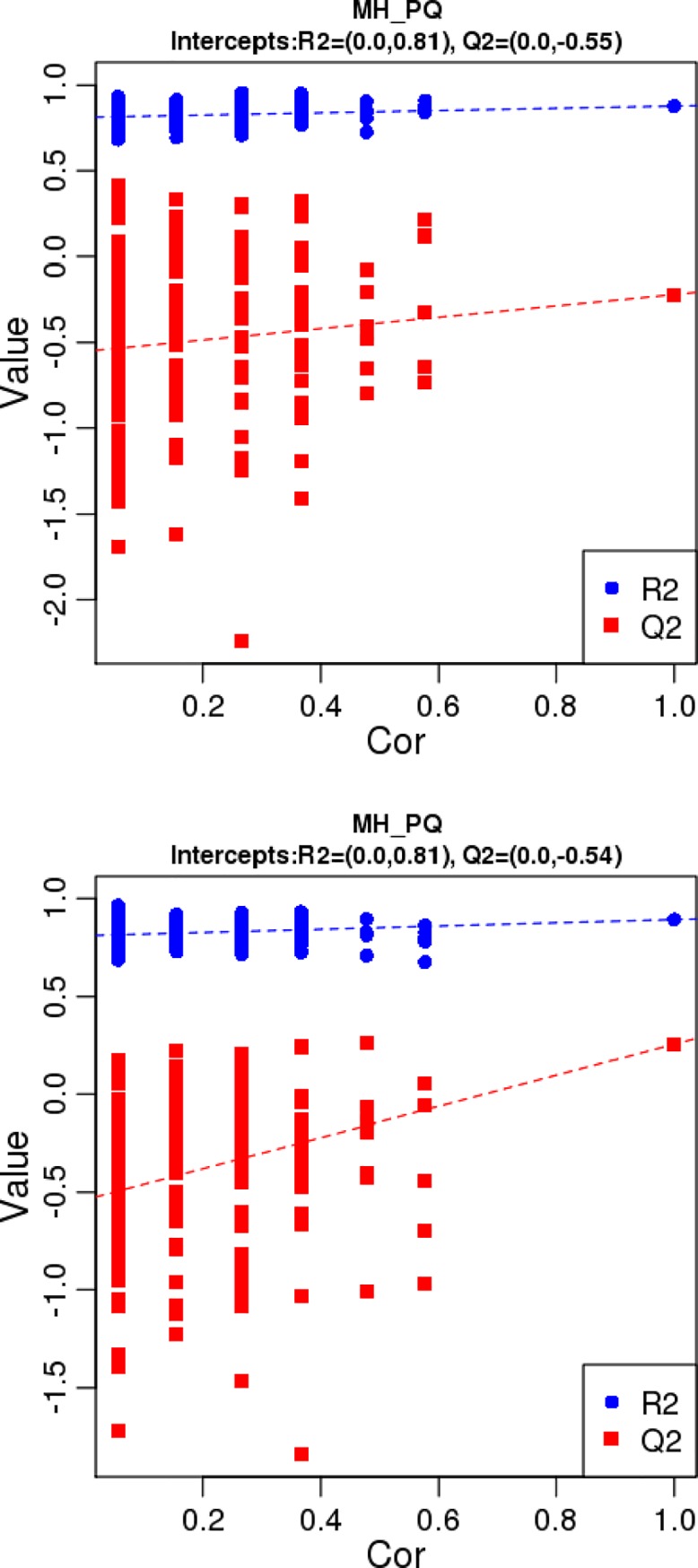
PLS-DA valid figure, the quality of the fitting model can be explained by R^2^ and Q^2^ values. R^2^ displays the variance explained in the model and indicates the goodness of fit. Q^2^ displays the variance in the data predictable by the model and indicates the predictability. The above figure represents the results were obtained under the MS positive ion mode, and the below figure represents the results were obtained under the MS negative ion mode.

PCA and partial least squares discriminant analysis (PLS-DA) were applied to obtain meaningful statistical results. The PC2 scores plot (Fig 4) showed a clear separation between the MH group and the PQ group, with a 95% confidence interval. PLS-DA was performed to further improve the separation. The plot of PLS-DA (Fig 5) also showed a clear separation between the MH and the PQ group. To demonstrate the reliability of the model, we applied PLS-DA to the concentration data of assigned metabolites (Fig 6). The quality of the fitting model was explained by R^2^ and Q^2^ values. R^2^ displays the variance explained in the model and indicates the goodness of fit. Q^2^ displays the variance in the data predictable by the model and indicates the predictability. The results showed that the model has good reliability and predictability.

**Fig 7:**
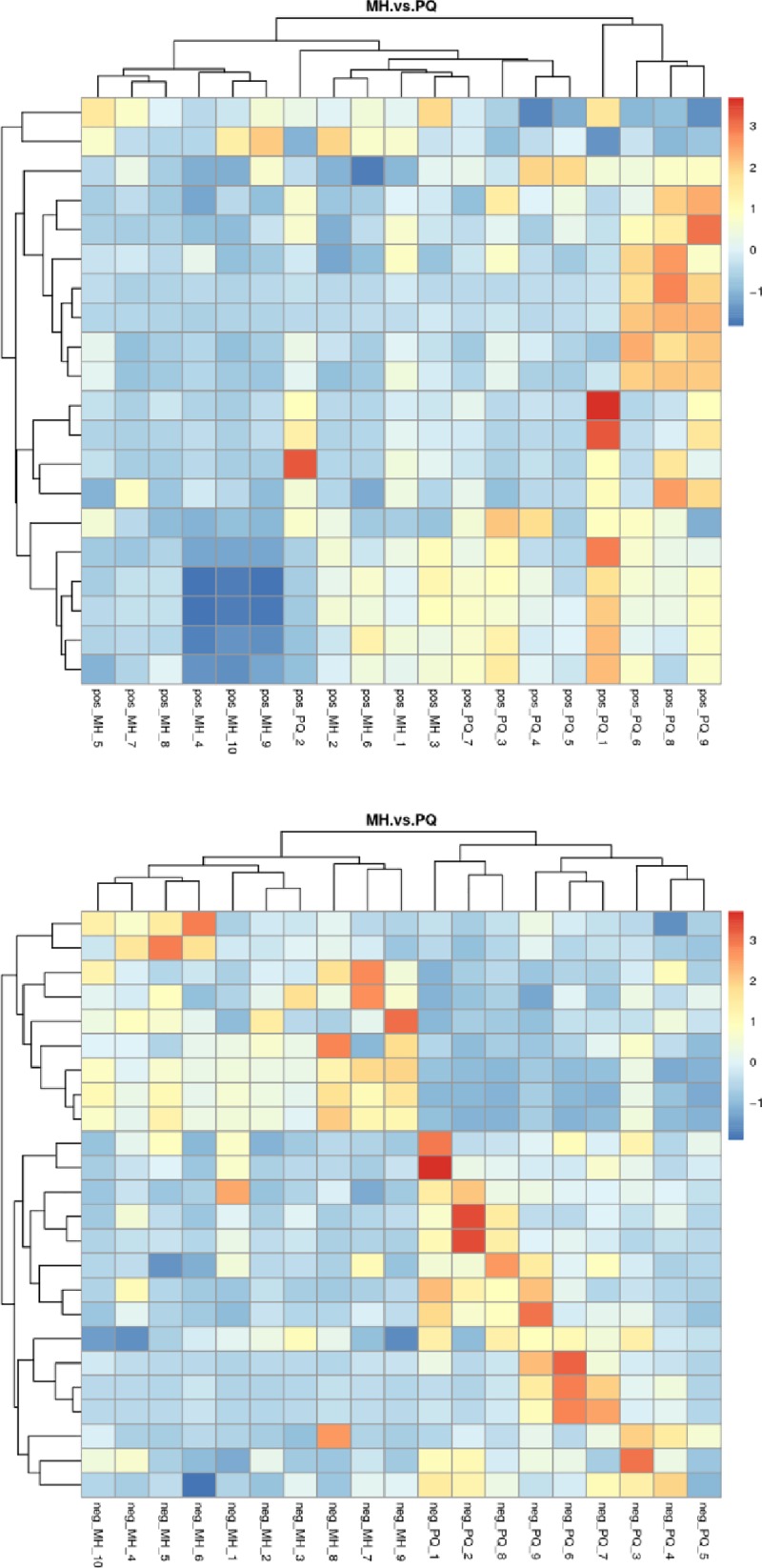
Heat map of differential metabolites cluster analysis, the above figure represents the results were obtained under the MS positive ion mode, and the below figure represents the results were obtained under the MS negative ion mode.

Cluster analysis or clustering is the task of grouping a set of objects in such a way that objects in the same cluster are more similar (to some extent) to each other than to those in other clusters. As shown in Fig 7, the PQ group and MH group showed a clear separation in the clustering analysis of metabolites.

**Fig 8:**
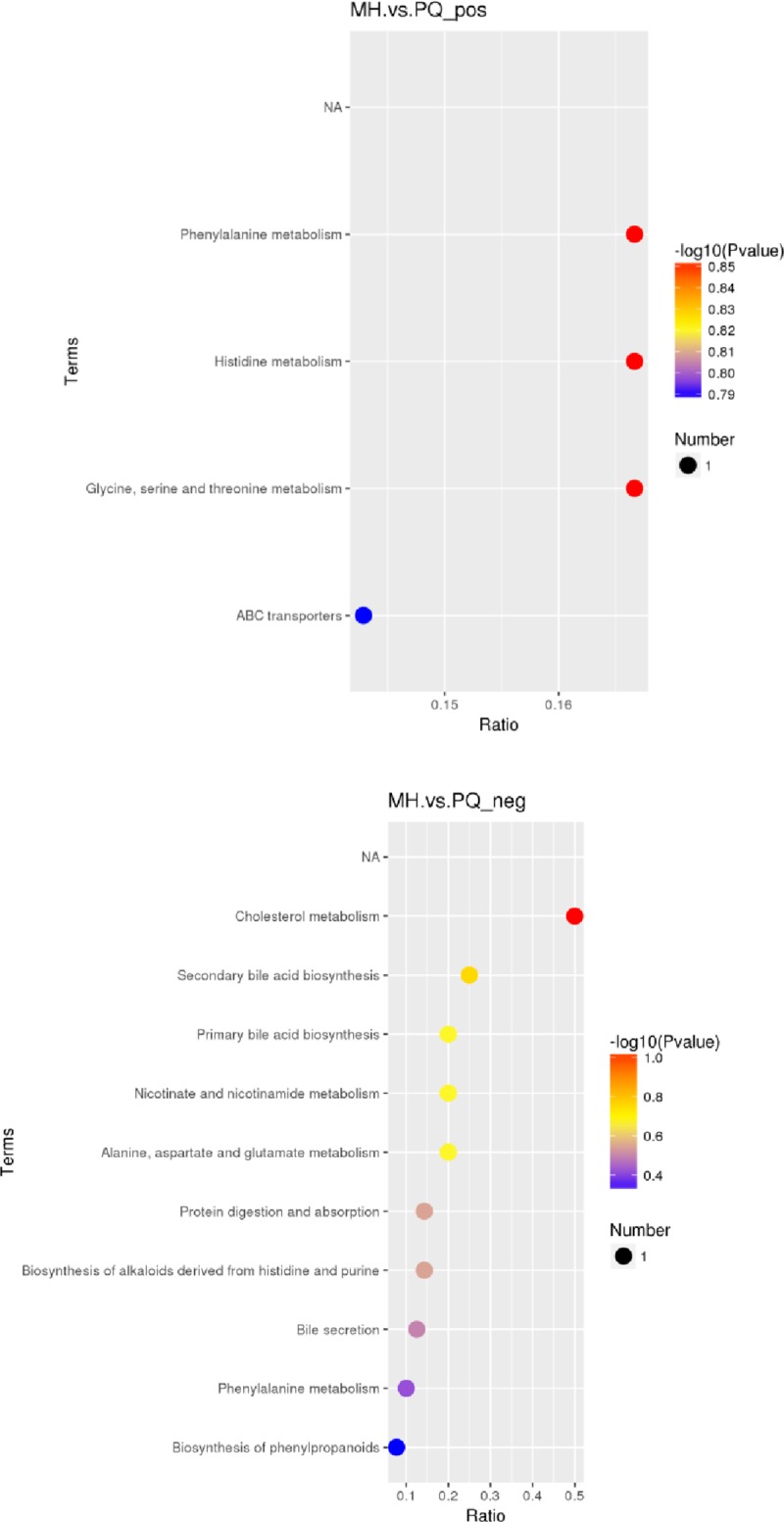
KEGG enrichment bubble chart (the upper figure represents the KEGG enrichment results under the MS positive ion mode, and the below figure represents the KEGG enrichment results under the MS negative ion mode. The abscissa in the figure is the ratio of the number of different metabolites in the corresponding pathway to the number of identified total metabolites. The higher the ratio, the higher the concentration of differential metabolites in the pathway. The color of the dot represents the p-value of the hypergeometric test. The smaller the p-value, the greater the reliability and the more statistically significant of the test. The size of the dot represents the quantity of differential metabolites in the corresponding pathway. A larger point size indicates more different metabolites in the pathway).

We identified the differential metabolites between the MH group and the PQ group (Table 2). As shown in Fig 8, these different metabolites mainly participate in metabolism of phenylalanine, histidine, glycine, serine, threonine, alanine, aspartate, glutamate, nicotinate, nicotinamide and cholesterol, as well as in bile acid biosynthesis in the KEGG enrichment bubble chart.

**Table 2:** The distinguished different metabolites between the MH group and the PQ group.

No.	name	PVALUE	ROC	VIP	Trend	Pathway
1	Betaine	0.0165039	0.822222222	2.333836932	down	Glycine, serine and threonine metabolism
2	1-Methylhistamine	0.017435099	0.822222222	1.845271752	up	Histidine metabolism
3	Hippuric acid	0.0440725	0.788888889	1.210346209	down	Phenylalanine metabolism
4	Taurochenodeoxycholic acid	0.035375931	0.777777778	3.430005021	down	Cholesterol metabolism
5	N-Acetyl-L-aspartic acid	0.033185023	0.755555556	1.130018742	up	Alanine, aspartate and glutamate metabolism
6	Maleamic acid	7.86E-06	0.966666667	3.031203723	up	Nicotinate and nicotinamide metabolism
7	3-Methylbutanoic acid	0.016541182	0.8	1.123873657	up	Protein digestion and absorption
8	Yangonin	0.020999492	0.833333333	2.236700181	down	Biosynthesis of phenylpropanoids
9	taurohyocholic acid	0.040083057	0.766666667	4.412830032	down	Taurine and hypotaurine metabolism

## Discussion

Although multiple organs are involved, the lung is the main target organ of PQ poisoning [30]. The early manifestation of PQ poisoning is acute lung injury, whereas progressive pulmonary fibrosis, a typical feature of PQ poisoning, develops in a later stage, thus leading to a poor prognosis [31,32]. At present, toxicological mechanisms of PQ are widely acknowledged to be free radical oxidative damage and mitochondrial damage. Therefore, inhibition of oxidative stress injury is an effective treatment for acute lung injury caused by PQ poisoning.

MH, an effective component in Rana Oviductus Ranae [33,34], has roles in protecting against infection and enhancing serum protein and anti-oxidation [33]; it can be used to treat inflammation, immune diseases and diseases caused by ROS and free radicals. In this experiment, there was no difference between the MH and PQ groups in the levels of SOD and LDH, but there was a clear difference between the PQ groups and the control group, and between the MH group and the control group. There was a significant difference between the MH and PQ group in the level of MDA, thus indicating that MH decreased the level of MDA. Moreover, the MDA levels in the MH and PQ groups were significantly lower than that in the control group. These results were consistent with traditional Chinese medicine, in which Oviductus Ranae is used to lower MDA levels and prevent free radical damage to the structure and function of endothelial cells [34]. MH decreases the expression of inflammatory cytokines, such as TNF-α. As shown in Fig 2, we speculated that MH may decrease inflammation in lung tissue after PQ administration.

PCA and PLS-DA indicated significant differences in metabolic patterns between the MH and PQ groups. The PLS-DA graph confirmed that the model has good stability and predictability. KEGG enrichment analysis revealed that the differences in metabolic patterns between the MH and PQ groups were mainly reflected in the decrease in the content of betaine, which participates in glycine, serine and threonine metabolism. The level of hippuric acid, which participates in phenylalanine metabolism, decreased in the MH group. The increase in N-acetyl-L-aspartic acid reflected the effects of MH on alanine, aspartate and glutamate metabolism. Moreover the increase in 3-methylbutanoic acid reflected that MH affects protein digestion and absorption. Overall, we speculate that the MH group was protected against lung toxicity caused by PQ poisoning through changes in amino acid metabolism patterns. The decrease in Yangonin and taurohyocholic acid indicated that MH affects the biosynthesis of phenylpropanoids, as well as taurine and hypotaurine metabolism.

The increased content of 1-methylhistamine in the MH group was consistent with the reported pharmacological effects of histidine supplementation in decreasing inflammation [35]. The metabolic differences between the MH and PQ groups were mainly concentrated in the metabolic pathways of nicotinate and nicotinamide metabolism, because the main toxicology mechanism of PQ poisoning is oxidative damage. On the basis of the differences in nicotinate acid and nicotinamide metabolism, after PQ administration, excessive ROS and reactive nitrogen species were produced, and nicotinamide adenine dinucleotide phosphate was consumed; thus, affecting the redox balance *in vivo* and resulting in body damage. In the MH group, the level of maleamic acid increased to protect against the toxicity of the PQ due to oxidation. In conclusion, the changes in the metabolic pattern were consistent with the anti-inflammatory and anti-oxidative pharmacological effects of MH.

The changes in the metabolic pattern in the MH group were also manifested in cholesterol metabolism, which showed a decrease in taurochenodeoxycholic acid, thus indicating perturbed cholesterol metabolism. MH appeared to correct the cholesterol metabolism disorder after the PQ poisoning.

In conclusion, we found that the metabolic patterns in the MH group and PQ+MH group were significantly different, as mainly reflected in the metabolic pathways of phenylalanine, histidine, threonine, glycine, serine, alanine, glutamic acid and asparagine; bile acid biosynthesis; and nicotinate and nicotinamide metabolism. In addition, MH decreased the levels of MDA and TNF-α after the PQ poisoning. Our results indicate that MH attenuates paraquat-induced acute lung injury possibly via antioxidant and anti-inflammatory mechanisms. This study is expected to improve the pharmacological effects of MH and provide new ideas for the management of PQ. However, the mechanism of MH requires further study.

## Supporting information

S1 File MH.vs.PQ-metexprquant. (ZIP)
