# Supplementary figures and images for: Protective mechanism of 1-methylhydantoin against lung injury induced by paraquat poisoning
Source: PLoS One. 2019 Sep 27;14(9):e0222521. doi: 10.1371/journal.pone.0222521 (PMC6764654; doi:10.1371/journal.pone.0222521)

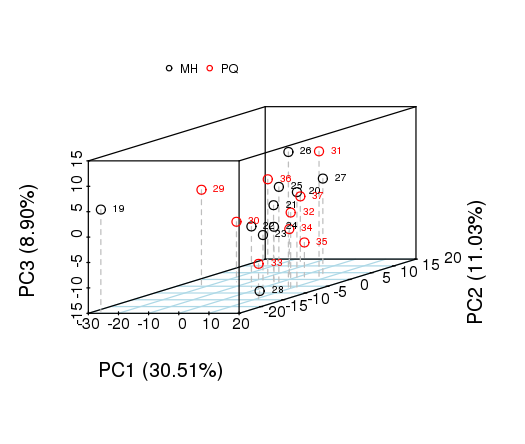

Supplement: S1 File — (ZIP) [file pone.0222521.s001.zip › MH.vs.PQ-metexprquant/MH.vs.PQ_neg_PCA.3D.png]

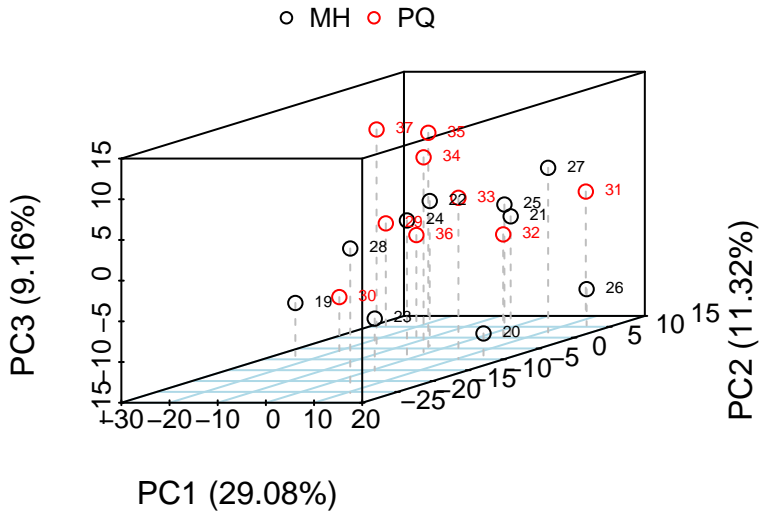

Supplement: S1 File — (ZIP) [file pone.0222521.s001.zip › MH.vs.PQ-metexprquant/MH.vs.PQ_pos_PCA.3D.pdf]

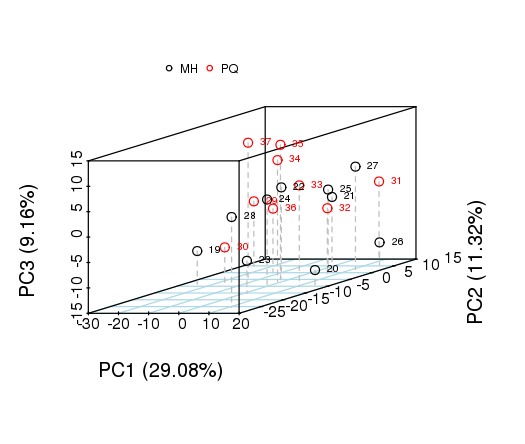

Supplement: S1 File — (ZIP) [file pone.0222521.s001.zip › MH.vs.PQ-metexprquant/MH.vs.PQ_pos_PCA.3D.png]

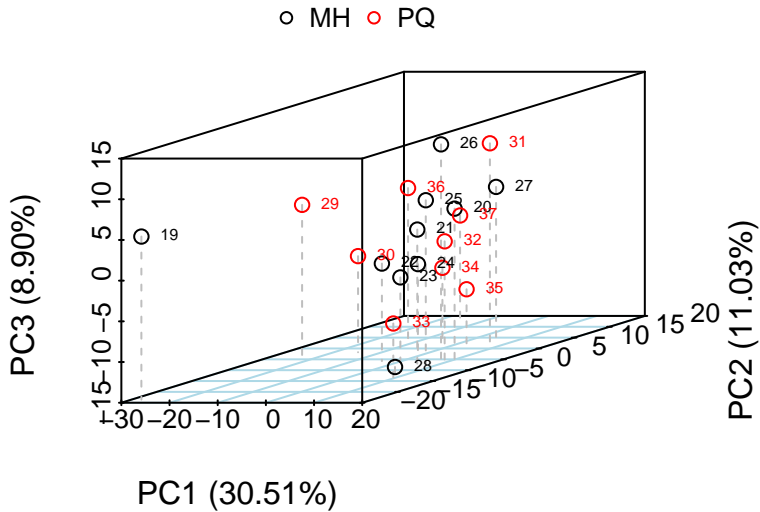

Supplement: S1 File — (ZIP) [file pone.0222521.s001.zip › MH.vs.PQ-metexprquant/MH.vs.PQ_neg_PCA.3D.pdf]
